# Predictors of caffeine consumption patterns in high school athletes

**DOI:** 10.1080/15502783.2025.2525378

**Published:** 2025-06-28

**Authors:** Eamonn M. O’Connell, Disa L. Hatfield, Amanda Stors, Steven A. Cohen

**Affiliations:** a University of Rhode Island, Department of Kinesiology, South Kingston, RI, USA; b University of Rhode Island, Department of Health Studies, South Kingston, RI, USA

**Keywords:** Caffeine, adolescents, athletes, supplements

## Abstract

Caffeine is the most widely used supplement by athletes. Caffeine’s benefits and its accessibility have led to increased consumption in young adults, despite limited research on athletic performance, efficacy, and possible side effects in this population. In addition, little research has been published examining the patterns of consumption in young athletes.

**Purpose:**

The purpose of this study was to examine caffeine consumption and the factors that affect caffeine use in high school athletes.

**Methods:**

Three hundred and ninety-four Rhode Island high school athletes (age: 16.8 ± 1.27 years) completed a cross-sectional online survey to assess caffeine consumption. A multivariate logistic regression analysis and binary logistic regression were performed to characterize associations between use and nonuse and the independent variables of ethnicity, grade, sex, and sport played. 95% confidence intervals (CI) were calculated for all models. Statistical significance was set to *p* ≤ 0.05 for all analysis.

**Results:**

Fifteen point five percent of the variance in caffeine use was related to ethnicity, grade, and sex(R^2^ = 0.155), with significant results for each variable. A majority of female athletes consumed caffeine (67.4%), compared to male athletes (37.8%) (*p* < 0.001). Athletes identifying as Caucasian reported significantly more caffeine use (55.9%), compared to athletes from underrepresented backgrounds (32.6%) (*p* < 0.001). Caffeine use was significantly different across grades, where 30.6% of 9th graders, 49.2% of 10th graders, 55.2% of 11th graders, and 49.2% of 12th graders reported caffeine use (*p* = 0.049). Coffee (51%) and energy drinks (43%) were the primary sources of caffeine consumption. Dance, cheerleading, and gymnastics participants were more than ten times as likely to consume caffeine compared to other sports (95% CI [2.28, 48.94], Exp (β) = 10.57).

**Conclusion:**

Caffeine use in young athletes is related to factors including sex and ethnicity. Given the prevalence of use, young athletes, coaches, and parents/guardians should be educated on the risks and benefits of caffeine use. Future research should focus on the potential benefits of caffeine use to athletic performance verses possible side effects in this population.

**Supported by:**

The Clean Competition Grant from the Rhode Island Foundation.

## 1. Introduction

Caffeine is one of the most widely used supplements globally [1]. Perhaps one of the most well-known uses of caffeine is that caffeine can be used to boost athletic performance [2]. For instance, caffeine has been shown to interact with the neuromuscular system and decrease certain inhibitory mechanisms of the central nervous system (CNS), which translates to increased sports performance [3]. Additionally, caffeine has various interactions with the primary energy systems of the body and can decrease glycogen utilization and increase fat oxidation, thus acutely increasing VO_2max_ in athletes, increasing an athlete’s endurance and overall energy output [4]. Caffeine has also been shown to decrease perceived exertion in sports, which allows athletes to continue play at higher intensities for longer durations [4,5]. These primary physiological benefits lead caffeine to be an ubiquitously used supplement for athletes of all types and of all skill levels, as well as being utilized on a regular basis for sports practice, competition performance, and daily life.

Caffeine is primarily taken orally, via drinks such as coffee, energy drinks, pre-workout supplements, tea, soda, and many more beverages. Digestion begins in the gastrointestinal tract, where caffeine is brought into the bloodstream and delivered into systemic circulation by passing through various cellular membranes, including the blood-brain barrier [6]. From circulation, caffeine interacts with the various systems that caffeine comes in contact with and has a multitude of results based on the type of tissue. These interactions give the user the typical benefits that caffeine is associated with such as staving off tiredness, increasing alertness, and increasing energy [2].

The benefits of caffeine supplementation have made caffeine a popular choice for athletes over the decades and has seen increased use in the adolescent athletic population. Unlike the adult population, the benefits of caffeine supplementation for adolescents are largely unknown, as few caffeine randomized control trials have been done [7–10]. Current research in adolescents suggests that caffeine supplementation, at various dosages, is associated with increased anxiety and irritability, decreased response time, decreased attention, temporary and reversible cardiovascular events [11,12]. These side effects can potentially decrease sports performance and cause negative health consequences outside of sports play [13].

Data from nationwide surveys in the United States from 2003 to 2012 indicate that 86–90% of US teenagers (13–17 years) consumed added caffeine sources (e.g. caffeinated beverages) in addition to intrinsic caffeine consumption (e.g. via foods such as chocolate) [14]. As of 2012, children ages 14–19 years consumed an average of 61 mg/day of caffeine, with the largest source being soda, followed by tea, and coffee [15]. Notably, in the adolescent general population, caffeine consumption has decreased from 1999–2000 to 2011–2012, with soda having the sharpest decrease in consumption over that period [15].

In addition to the scarcity of data on caffeine’s effect on adolescents, there is no current information for the adolescent athletic population. Understanding the patterns of caffeine use and the factors which impact caffeine consumption in adolescent athletes distinct to those patterns and impacts in other age groups are integral for educating parents, coaches, health professionals, and all other caregivers on the risks and benefits of caffeine consumption. From this information, these caregivers may be able to make more informed decisions regarding adolescent caffeine supplementation. Therefore, the purpose of this study was to examine caffeine use among high school athletes and the factors that affect caffeine use, such as sex, age, and ethnic/cultural background. The authors hypothesized that significant patterns would be present in caffeine consumption in young athletes for one or more demographic variables including age, sex, ethnic background, sport, and socioeconomic status.

## 2. Methods

### 2.1. Participants

Three hundred and ninety-six Rhode Island high school athletes (age: 16.8 ± 1.27 years) completed a cross-sectional online survey to assess their supplement and caffeine usage. Individuals had to be between the ages of 14 and 19 years old, live in the state of Rhode Island, and participate in interscholastic sports in Rhode Island. Exclusion criteria included participants who did not currently live in the state of Rhode Island, were not enrolled in high school, were enrolled in college, and if they did not play a sport/were sedentary. Demographic information and sports participation breakdown can be found in Tables 1 and 2, respectively.

**Table 1.** Demographic information.

Demographic Information	*n*
Sex	
Male	252
Female	139
Other/Prefer not to answer	3**
Age (Years of age)	
14	20
15	51
16	70
17	97
18	138
19	12
Prefer not to answer	6
Grade	
9th	49
10th	59
11th	87
12th	195
Prefer not to answer	6
Ethnicity/Cultural Background	
White or Caucasian	256
Black or African American	40
Hispanic, Latino, or Spanish Origin	43
Asian	26
Other Race, Ethnicity, or Origin*	41

*American Indian, Middle Eastern, Somalian, prefer not to Answer. **Participants not included in statistical analyses.

**Table 2.** Sport participation breakdown.

Sport	Male Athletes (n)	Female Athletes (n)
Volleyball	4	15
Swimming	8	10
Basketball	74	35
Football	63	0
Track & Field	49	18
Wrestling	25	2
Cross Country	38	18
Lacrosse	27	51
Soccer	71	47
Baseball/Softball	36	23
Field Hockey	1	7
Tennis	31	9
Ice Hockey	21	8
Dance/Cheerleading/Gymnastics	0	18
Golf	1	9
Other*	17	3

*Other = Sailing, Rugby, Gym, Water Skiing, Martial Arts, Jiu-jitsu, Boxing, Fencing, Rowing, Squash, Weight Training/Weightlifting, Ping-Pong, Ski.

### 2.2. High school athlete supplement knowledge (H-ASK) questionnaire

This study was part of a larger survey examining knowledge and patterns of supplement consumption in teenage athletes. Participants were queried about demographical information, caffeine usage, and other questions pertaining to caffeine use and supplementation. Frequency (i.e. how often the participant was taking caffeine or the relevant supplement) and duration (i.e. how long the participant had been taking caffeine or the relevant supplement for) information was also collected.

### 2.3. Procedures

Participants were asked to complete the High School Athlete Supplement Knowledge questionnaire (H-ASK) via Qualtrics (Qualtrics, Provo, Utah). Recruitment was completed by word of mouth and participants were provided a link to the survey or a QR code on the associated flier. Prior to starting the questionnaire participants read and signed an informed assent/consent, depending on their age. Parental consent was waived by the Institutional Review Board. The H-ASK survey data was de-identified and stored on a secure computer at the University of Rhode Island. This study was conducted with approval from University of Rhode Island Institutional Review Board (protocol# I2021–176) and was performed in accordance with the principles stated in the Declaration of Helsinki.

Consequent to opening the URL or scanning the QR code on the associated flier, participants were directed to the Qualtrics online survey. Before completing any questions, participants were required to read and sign an informed consent/assent, depending on their age. At the end of the informed consent/assent participants had the option to click yes, meaning they do provide consent/assent, or no, meaning they do not provide consent/assent. If the participant selected no to consent/assent, they were directed to the end of the survey where they provided their name and e-mail address to enter a drawing for a $40 Amazon gift card for their time. If the participant selected yes to the consent/assent, they were directed to the start of the H-ASK questionnaire survey. A similar process for entering the drawing was done for those who selected yes to the informed consent/assent, however this occurred after completing the H-ASK questionnaire completely.

### 2.4. Statistical analyses

For the purposes of running various statistical analyses with more robust cell sizes, several groups of variables were combined into a single category. Regarding sports, the following were combined into a single category: “dance,” “cheerleading,” and “gymnastics.” A separate category was created to include “other” sports, which included “sailing,” “rugby,” “gym,” “water skiing,” “martial arts,” “jiu-jitsu,” “boxing,” “fencing,” “rowing,” “squash,” “weight training/weightlifting,” “ping-pong,” and “skiing.” For caffeine sources, the category “energy drinks” was created to include the following: “Celsius,” “Alani Nu,” “C4,” “Redbull,” “Monster,” “Bang,” and “Reign Energy.” Caffeine pills were omitted from statistical analysis as there were only two responses reporting using them, and caffeine pills are a drastically different method of caffeine ingestion from the other responses. For reported caffeine frequency, the following categories were added to the “a few times a month” category, including “rarely,” “once a month,” “2–3 times a week,” “a few times a year,” and “maybe 1–2 times every 6 months.” Lastly, for reported caffeine duration, all responses that were reported as less than a year in duration were combined as “less than a year,” including: “1–3 months,” “4–5 months,” “6–12 months,” “less than 1 month,” “once,” “almost never,” “rarely,” “I have only used energy drinks a couple times before games,” and “not recently.”

Chi-square analyses were conducted to compare caffeine consumption across grades, age, sport, caffeine frequency and duration between sexes, and caffeine product consumption and sex. Logistic and binary regression models were used to assess associations between caffeine use and independent variables that included ethnicity, grade, sex, and sport played. A value of *p* ≤ 0.05 was set as statistical significance for all analysis, and 95% confidence intervals (CI) were calculated for all odds ratios obtained in the logistic regression models.

## 3. Results

Regarding differences in caffeine use among ethnicities, white or Caucasian participants reported significantly greater caffeine use (*n* = 151, 53.2%) compared to non-use (*n* = 133, 46.8%; *p* < 0.001). Additionally, Asian participants reported significantly less caffeine use (*n* = 6, 23.1%) compared to non-use (*n* = 20, 76.9%; *p* = 0.023). There were no significant differences between caffeine use and non-use in Black, American Indian, Middle Eastern or North African, and Hispanic, Latino, or Spanish Origin participants (*p* > 0.05).

The reported frequency of caffeine consumption between male and female athletes varied significantly. There was a significant difference between male athletes (*n* = 18; 48.6%) and female athletes (*n* = 19; 51.4%; *p* = 0.042) who reported consuming caffeine daily. Female athletes (*n* = 34; 54%) reported a significantly greater frequency of consuming caffeine 2–6 times per week compared to male athletes (*n =* 29; 46%; *p* = 0.001). However, male athletes (*n=* 27; 50.9%) reported consuming caffeine “a few times a month” significantly more than female athletes (*n* = 26; 49.1%; *p* = 0.034). There was no significant difference between male (*n* = 5; 45.5%) and female athletes (*n* = 6; 54.5%; *p* = 0.195) who reported consuming caffeine more than once per day. Lastly, there was no significant difference between male (*n* = 14; 66.7%) and female athletes (*n=* 7; 33.3%; *p* = 0.791) who reported having used caffeine in the past, but not currently.

The duration of caffeine consumption among those who consume caffeine was significantly different between sexes for those that reported caffeine use over a year in time (*p* < 0.001). Sixty-five female athletes participants (54.2%) reported consuming caffeine for over a year, compared to 55 male athletes participants (45.8%). There was no significant difference between male (*n* = 38; 58.5%) and female athletes (*n* = 27; 41.5%; *p* = 0.312) for those that reported caffeine consumption under a year. More information on caffeine consumption duration can be found in Table 3.

**Table 3.** Sex differences in caffeine consumption frequency and duration.

	Male (*n)*	Female (*n)*	*p*-value
Caffeine Frequency			
More than Once a Day	5 (2.0%)	6 (4.3%)	<0.001*
Daily	18 (7.1%)	19 (13.5%)	
2 to 6 Times per Week	29 (11.5%)	34 (24.1%)	
A Few Times a Month	27 (10.7%)	26 (18.4%)	
Used in the Past	14 (5.6%)	7 (5.0%)	
Never Used	159 (63.1%)	49 (34.8%)	
Caffeine Duration			
Over a Year	55 (45.8%)	65 (54.2%)	<0.001*
Under a Year	38 (58.5%)	27 (41.5%)	

*Significant difference between male and female athletes (*p* ≤ 0.05).

Various significant interactions were found regarding age and grade. Less than half of 9th-grade athletes reported consuming caffeine (*n* = 15, 30.6%; *p* = 0.009). Conversely, 10^th^-grade athletes (*n* = 29, 49.2%), 11th-grade athletes (*n* = 48, 55.2%), and 12^th^-grade athletes (*n* = 96, 49.2%) reported greater likelihood of caffeine consumption. However, in 10th- (*p* = 0.858), 11th- (*p* = 0.133), and 12th-grade athletes (*p* = 0.650), caffeine consumption was not significantly different across all grades. When examining age and caffeine use independently, significant differences are present between ages (*p* = 0.003), with varying percentages reporting caffeine use, as follows: 14 years of age (55%), 15 years of age (29.4%), 16 years of age (50%), 17 years of age (55.7%), 18 years of age (47.1%), and 19 years of age (66.7%).

The primary source for caffeine consumption between male and female athletes was coffee (*n* = 117), with female athletes (*n* = 71, 60.7%) consuming significantly more coffee than male athletes (*n* = 46, 39.3%, *p* < 0.001). Following coffee, energy drinks (*n* = 100) were the second most popular option with male (*n* = 60, 60%) and female athletes (*n* = 40, 40%); however, there were no significant differences in energy drink consumption between either group (*p* = 0.320). There were no significant differences in soda or tea use between male and female athletes. A full report of caffeine source consumption can be found in Table 4.

**Table 4.** Sex differences in caffeine product consumption frequencies.

Caffeine Product	Male (*n)*	Female (*n)*	*p*-value
Energy Drinks	60	40	0.320
Coffee	46	71	<0.001*
Tea	2	4	0.113
Soda	5	1	0.323
Caffeine Pills	2	0	0.289

*Significant difference (*p* ≤ 0.05).

Across all participants, 15.5% of variance in overall caffeine use was related to the individual’s ethnicity, grade, and sex based on Nagelkerke’s R^2^ (R^2^ = 0.155), which relates to a medium-large effect size [16]. Significant results can be seen for female adolescent athletes (Exp (β) = 2.842; 95% CI [1.792, 4.508]), 11^th^-grade adolescent athletes (Exp (β) = 2.627; 95% CI [1.202, 5.740]), and Asian adolescent athletes (Exp (β) = 0.308; 95% CI [0.115, 0.822]). Table 5 has more information regarding the demographic logistic regression analysis.

**Table 5.** Binary logistic regression for demographic determinants of caffeine use.

Covariate	Exp β	95% CI [Lower, Upper]	*p*-value
Sex			
Female	2.842	[1.792, 4.508]	<0.001*
Ethnicity			
Black	0.554	[0.267, 1.148]	0.112
Hispanic	0.575	[0.282, 1.173]	0.128
Asian	0.308	[0.115, 0.822]	0.019*
Other†	0.855	[0.413, 1.769]	0.673
Grade			
10^th^	2.184	[0.933, 5.113]	0.072
11^th^	2.627	[1.202, 5.740]	0.015*
12^th^	1.910	[0.943, 3.870]	0.072

*Significant difference (*p* ≤ 0.05).

Other race, ethnicity, or origin: American Indian, Middle Eastern, Somalian, Prefer not to Answer.

With respect to the logistic regression for caffeine and sport participation, 17.7% of variance in caffeine use was related to the sport the individual played, based on Nagelkerke’s R^2^ (R^2^ = 0.177), which relates to a medium-large effect size [16]. Significant results can be seen for football (Exp (β) = 0.358; 95% CI [0.186, 0.691]), lacrosse (Exp (β) = 2.809; 95% CI [1.579, 4.999]), baseball/softball (Exp (β) = 1.953; 95% CI [1.034, 3.687]), dance, cheer, and gymnastics (Exp (β) = 10.568; 95% CI [2.282, 48.941]), and other sports (Exp (β) = 3.022; 95% CI [1.095, 8.346]). Table 6 has more information regarding the sport’s logistic regression analysis.

**Table 6.** Logistic regression for sports determinants of caffeine use.

Covariate	Exp β	95% CI [Lower, Upper]	*p*-value
Sport			
Volleyball	2.296	[0.789, 6.676]	0.127
Swimming	1.343	[0.492, 3.665]	0.565
Basketball	1.058	[0.640, 1.748]	0.826
Football	0.358	[0.186, 0.691]	0.002*
Track and Field	0.803	[0.412, 1.563]	0.518
Wrestling	0.847	[0.357, 2.012]	0.708
Cross Country	1.019	[0.506, 2.051]	0.958
Lacrosse	2.809	[1.579, 4.999]	<0.001*
Soccer	1.232	[0.743, 2.043]	0.419
Baseball/Softball	1.953	[1.034, 3.687]	0.039*
Field Hockey	0.206	[0.038, 1.114]	0.067
Tennis	0.573	[0.281, 1.171]	0.127
Ice Hockey	0.852	[0.363, 2.002]	0.714
Dance, Cheer, Gymnastics	10.568	[2.282, 48.941]	0.003*
Golf	0.815	[0.216, 3.070]	0.762
Other†	3.022	[1.095, 8.346]	0.033*

*Significant difference (*p* ≤ 0.05).

†Sailing, Rugby, Gym, Water Skiing, Martial Arts, Jiu-jitsu, Boxing, Fencing, Rowing, Squash, Weight Training/Weightlifting, Ping-Pong, Ski.

## 4. Discussion

The objectives of this study were twofold: to understand the caffeine use among Rhode Island adolescent athletes, and to evaluate the factors that potentially influence caffeine use the most in this population. The author’s hypothesis that significant patterns of caffeine consumption would be related to one or more demographical variables was validated. The major findings from this study are that adolescent athletes are using caffeine, and the primary factors that impact adolescent caffeine use are sex, ethnicity, grade, and sport played. Regression analysis completed regarding ethnicity, grade, sex, and sport played indicated that sex and ethnicity had the highest correlation to caffeine use among adolescent high school athletes.

Various sex differences were noted in the results, including caffeine consumption frequency and duration. Female high school athletes reported significantly greater daily and weekly caffeine consumption. This can potentially be linked to the difference in sports type, where young female athletes tend to participate in sports with longer practice/competition times, such as cheerleading, dance, and certain track and field events [17]. When examining the logistic regression for sports determinants of caffeine use, it was made apparent that female-dominant sports including dance, cheer, gymnastics had much greater odds for consuming caffeine. In fact, dance, cheerleading, and gymnastics, which was exclusively a female sport (see Table 2), were 10.56 times as likely to consume caffeine compared to other sports. Similarly, lacrosse players for this sample were predominantly female (see Table 2) and indicate similar findings, where lacrosse players were 2.81 times as likely to consume caffeine compared to other sports. In contrast to these female dominant sports, football players, a male dominant sport (see Table 2), was significantly less likely to consume caffeine compared to other sports.

Female-dominated sports such as gymnastics, dance, and cheerleading often have longer practice times. This may pose additional perceived and actual fatigue to these athletes and require caffeine to reduce the feeling of fatigue. These female athletes may be using caffeine daily and/or weekly to maintain higher energy levels for the demand of their sports and schooling. As reported by Ludden and colleagues, the primary use for caffeine in high school students is for reducing symptoms of fatigue as well as perceived improvements in sports performance [18,19]. An additional demand of some sports such as gymnastics, cheerleading, and dance is the lack of seasonality. The lack of structured seasons mayprovide pose additional fatigue compared to traditional seasonal sports in which there may be reduced training time.

The primary sex differences regarding duration of caffeine consumption demonstrated that young female high school athletes reported significantly greater caffeine use for a year or longer compared to male athletes. In addition to sport participation, another potential reason for longer caffeine use could be due to social interactions, where high school students reported consuming caffeine in social situations with their family, friends, and coworkers/classmates [18,20]. There may be an element of social status with caffeine use among high school students, where drinking caffeine products emulates older peers (such as older siblings, family members, etc.) and is linked to social status [18,20]. Because of this socio-cultural connection to caffeine use, female athletes may be exposed to caffeine consumption earlier, thus persisting for durations of one year or greater.

The differences in caffeine sources were minimal between sexes. There is currently minimal literature available on the differences between gender/sex regarding caffeine dietary sources, and to the authors knowledge, nothing available for an athletic population. National Health and Nutrition Examination Survey (NHANES) data taken from 2011 to 2012 regarding sources of caffeine in US children and adults indicated that primary sources of caffeine consumption in US youth ages 14–19 years old were as follows: soda (33%), tea (28%), coffee (25%), energy drinks (10%), and the remainder from milk and other beverages [15]. The results reported by Drewnowski and Rehm differ from those in this study, where coffee accounted for 50% of dietary caffeine intake, and energy drinks contributed 43% of dietary caffeine intake for adolescent athletes. The paucity of information in this regard, emergent findings, and contrasting findings from national survey data may indicate that further examination on the differences in caffeine source consumption in an adolescent athletic population is required before any conclusions are drawn on the subject.

Although there are no known studies examining the direct relationship between caffeine consumption and ethnicity in the adolescent athletic population, the results from this study reflect that of an adult population. Lieberman and colleagues reported that ethnicity was a significant factor in adult caffeine consumption, and non-Hispanic white adults consumed significantly more caffeine compared to nonwhite adults [21]. The data from Lieberman and colleagues, drawn from NHANES, encompasses a substantial sample size (*n* = 16,173) of US adults. This comparison strongly suggests that if this data pattern persists in the current study, it would yield comparable results in the adolescent athletic population [21]. Thus, it is further affirmed that the influence of ethnicity on caffeine consumption is the same in an adult population as an adolescent population and is likely rooted in socio-cultural ties.

As an additional note, logistic regression analysis indicated that Asian adolescent athletes were significantly less likely to consume caffeine compared to white adolescent athletes. Although this information affirms the above statements that white adolescent athletes consume more caffeine than nonwhite adolescent athletes on average, this was the only significant negative relationship indicated with caffeine use and demographic information. Further research may be warranted to understand the relationship between specific ethnic backgrounds and caffeine consumption in adolescent athletes.

In relation to age, there appears to be an apparent link with early caffeine use, and caffeine use throughout a lifetime, specifically from teenage years into adulthood. This association between teenage use and adult use is also reported by Tran and colleagues, utilizing NHANES data on United States teenagers and young adults, demonstrating that early caffeine use persists into adulthood [14]. In addition to this association of caffeine use and age; sex and ethnicity were closely linked to caffeine use and is further confirmed by use seen in adult populations via similar NHANES data [21].

These patterns of increasing caffeine use from adolescents into adulthood also tracks with previous research and may suggest a socio-cultural component that may affect this linear rise in caffeine consumption across maturation [14]. Socio-culturally, as the child gets closer to age as their adult peers, they may be more interested in using caffeine, to appear older in age, and thus a higher social status [18,20]. To further the point, commonly reportedsocio-cultural reasons for high school students consuming caffeine, as reported by Canadian high school students, include parental role modeling, image enhancement, and social norms [20]. Additionally, 9th-grade students may also not yet be involved as much in social situations where caffeine would be used, such as after school before after school activities, as was reported before [18].

One could also speculate that 9th-grade athletes may lack the amount of fatigue created from sports demand, unlike their 10th-, 11th-, and 12th-grade counterparts. The physical demands of sports during 9th grade are potentially much lower than that of a 12th-grade athlete, reducing the need for caffeine to reduce symptoms of fatigue. However, few studies have examined the direct link between grade and sports on fatigue levels in adolescent athletes, and studies that have done so indirectly could not draw strong conclusions [22]. Additionally, other studies have shown similar findings, where caffeine use increases linearly from early teenage years and spikes around 25–29 years of age [14,23]. The possibility exists that 9th-grade students do not yet have the same academic rigors that higher grade levels have, in addition to the rigors of higher-level sports.

While there was minimal variation between all grades, the logistic regression analysis indicated that 11th-grade athletes had 2.6 times greater odds of consuming caffeine compared to other grades. It is commonly known that around 10th- to 11th-grade high school–aged individuals can receive their provisional license and begin to drive motor vehicles and increase their autonomy. Being able to drive would greatly increase the adolescent athlete’s ability to purchase and consume caffeine on their own and without potential adult supervision, which could increase their likelihood of use. This concept could also increase the likelihood of social gatherings centered around caffeine use after school hours and before athletic events or practices [18]. More research should be done to explore the concept of emerging autonomy in high school athletes and how it may impact caffeine use.

In many cases youth inherently parallel many aspects of their parents/guardians and other influential figures, caffeine use is no exception to this phenomenon [24]. As many adults use caffeine for reducing feelings of tiredness and lack of alertness, so do adolescent [18]. The same is true for sports performance, as many adult athletes utilize caffeine supplements for their acute increases in sports performance. With the increase in the number of methods for caffeine supplementation, including beverages, gum, pills, candies, etc., it has become easier than ever for individuals to acquire and consume caffeine [24]. Understanding how often, and how much caffeine adolescent athletes consume can give parental and influential figures in the adolescent athlete’s life insight on whether concern and caution are needed for the athletes.

Understanding the amount and type of supplement used can help establish why these adolescent athletes are choosing to use a supplement. Participants reported that the primary reason for choosing to take a supplement was to increase energy (17.3%), which may explain why caffeine use was relatively high in this data set and why they were choosing to use supplements. When comparing this data to information conveyed in a focus group study done with young adolescents in Australia (*n* = 40, age = 12–15 years old), the overlapping themes were that youth in both studies reported using caffeine to increase energy and not feel as tired for school or sports [24]. In addition to the socio-cultural aspects of caffeine use, this may explain youth decide to supplement with caffeine.

Potential limitations for this study were minimal. However, it is worth noting that some interpretation of information was needed regarding certain survey results. The population of high school students may not have had much prior experience with surveys before, thus some open-ended responses required recategorization to run statistical analysis on. However, with the robustness of some answers provided, the open-ended responses allowed the researchers to truly understand the information that these high school students may know, as well as their understanding of what products do and do not contain caffeine. The recategorization did not impact the internal validity of this study and did not significantly impact the results and conclusions drawn. For further clarification on this, please refer to the statistical analysis section. Non-binary or other-prefer not to answer particpants were not included in the analyses due to the low number and statistical difficulties comparing groups with extreme differences in numbers. However, we chose to include this group in the descriptive information for transparency in inclusivity. To our knowledge, there is no research in this area describing a non-binary, other or trans-identifying athletes in the high school poulation.

Future research regarding adolescent athletes and caffeine use should focus on the dose response of caffeine in this population. As has been well reported now, adolescent athletes are drinking caffeine at increasing levels, and research needs to be done to provide information for guidelines on safe consumption of caffeine products for this population. Research indicates that developing brains are especially sensitive to caffeine; thus, the recommendations for adults may be drastically different for adolescents and teenagers [25]. This increase in sensitivity may render adolescents more susceptible to neurological and cardiac toxicity from caffeine, further warranting caution and additional analysis [26]. Additionally, understanding how caffeine impacts brain development and homeostasis of a developing individual may provide additional context for this topic
